# Exposure Assessment of Indoor PM Levels During Extreme Dust Episodes

**DOI:** 10.3390/ijerph17051625

**Published:** 2020-03-03

**Authors:** Itzhak Katra, Helena Krasnov

**Affiliations:** Department of Geography and Environmental Development, Ben-Gurion University of the Negev, Beer-Sheva 8410501, Israel; krasnovh@post.bgu.ac.il

**Keywords:** PM, dust events, indoor, outdoor

## Abstract

Millions of people live in areas that are subject to frequent dust events; however gaps remain in our knowledge about the association between dust, air quality and corresponding particulate matter (PM) exposure levels inside buildings. This case study demonstrates how the PM_2.5_ and PM_10_ levels in an urban environment respond to strong natural dust episodes. Real-time measurements were recorded simultaneously in indoor and outdoor environments in households in the city of Beer-Sheva, Israel during several strong dust events. A typical strong event was used for a detailed analysis of PM_10_ and PM_2.5_. Outdoor daily concentrations were above 1000 µg m^−3^ for PM_10_, the maximum hourly value of which was 1320 µg m^−3^. The indoor PM_10_ peaked at about 700 µg m^−3^ and fluctuated in parallel with the outdoor level but with a time lag of about 15 min. Indoor air tended to remain for several hours after the dust event had subsided. Analyses of multiple events revealed that the dependence of indoor PM_2.5_ and PM_10_ on natural dust varies but is not directly linked to the level of atmospheric dust concentration. From a health perspective, the exposure risk posed by extreme indoor PM_2.5_ and PM_10_ levels generated by natural dust episodes should be considered.

## 1. Introduction

Atmospheric dust from soil sources (dust events) plays an important role in the earth system [1], and as such, the process must be quantitatively understood from a variety of perspectives ranging from climate research (e.g., the source areas of wind-driven soil erosion) to human health. In terms of human health, viable health risk assessments must incorporate information about particulate matter (PM) and its chemical and biological properties [2]. Such exposure can have significant impacts on human health in these areas [3,4,5,6,7]. PM exposure data for epidemiological studies are derived from environmental monitoring of outdoor ambient levels that have been scrutinized intensely by regulatory agencies and health assessment panels.

In the immediate wake of a strong dust event, atmospheric PM_10_ concentrations can reach or exceed 1000 µg m^−3^ [8]. The guideline of WHO (World Health Organization) is a 24 h mean of 50 µg m^−3^ for PM_10_ (and 25 µg m^−3^ for PM_2.5_). The national guideline by the Israeli Ministry of Environmental Protection is 150 µg m^−3^ for PM_10_ only. The Ministry of Environmental Protection provides national alerts before dust storms with recommendations to limit outdoor activities and to stay inside buildings. However, our knowledge about the impact that such strong dust storms have on atmospheric PM_10_ in urban environments, particularly about the subsequent exposure levels indoors, where people spend most of their time, is clouded with uncertainty. Recent studies indicated that indoor PM levels may possibly be enhanced by even low atmospheric concentrations of less than 100 µg m^−3^ [9,10]. In addition, atmospheric dust was shown to be a major controlling factor for indoor PM_2.5_ and PM_10_ levels in an arid zone [10]. However, the impact of PM_2.5_ and PM_10_ levels generated from strong events on indoor levels remains unknown, although such events are common in the Mediterranean region [11,12].

Studies in the eastern Mediterranean have shown evidence of increases in the strength and frequency of Saharan dust-raising activity [13]. Moreover, a study by Krasnov et al. [8,14] of atmospheric PM_10_ levels revealed that the strongest storms in that region have occurred over the last few years. Several studies conducted in the Northern Negev (Israel) have shown the impact of dust- PM_10_ on public health, including asthma in children [3], cardiovascular morbidity [4], effects on serum glucose levels [15], onset of atrial fibrillation [16], and exacerbation of chronic obstructive pulmonary disease (COPD) [17].

There is growing concern about the potentially detrimental impact that dust pollution has on human health. Drylands (arid and semi-arid zones) that are subject to frequent dust events constitute about 40% of the Earth’s total land surface and contain an estimated 2.1 billion people. The exposure to extreme PM_2.5_ and PM_10_ levels due to natural dust episodes may cause or aggravate health effects. Little is known yet on the impact of dust storms on PM levels inside houses. This research paper describes a case where PM_2.5_ and PM_10_ levels in an urban environment respond to strong natural dust episodes while hypothesizing that indoor PM_2.5_ and PM_10_ concentrations can be very responsive. Real-time analyses enabled us to explore strong dust episodes in an urban environment located along the margin of the global dust belt and to assess their immediate impacts on outdoor and indoor PM_2.5_ and PM_10_ levels.

## 2. Methodology

**Figure 1:**
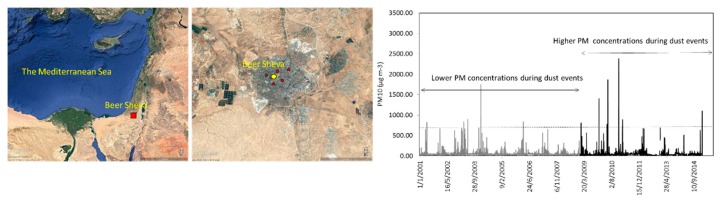
The study site and average daily concentrations of outdoor PM_10_ (µg m^−3^) over the last 15 years (1 January 2001–15 October 2015) recorded in the Beer-Sheva monitoring station. Dashed line represents strong events (>661 µg m^−3^) based on class levels by Krasnov et al. [8]. The locations of the households (red dots) and the monitoring station (yellow dot) are presented on the map.

Experimental site. The city of Beer-Sheva is located in the Negev desert region of southern Israel (Figure 1), and is suitable to achieve the aim of this research, namely, to assess the changes that occur in urban PM levels in response to strong natural dust episodes from distal and local sources [2,18]. Beer-Sheva frequently experiences dust storms whose durations are typically several hours to a few days [8]. Regional dust storms occur mostly during the winter, and are associated with the passage of cold fronts that are followed by periods with relatively high wind speeds (>6 m s^−1^). In contrast, the spring and the autumn are characterized by mild storms while the summer period is considered a dust-free season.

Environmental data. The distribution of the dust events over time corresponding to the indoor measurements we conducted is presented in Figure 1. Atmospheric PM_10_ data for 2001–2015 were obtained from a Ministry of Environmental Protection (http://www.sviva.gov.il) monitoring station located in the Negev desert that is part of the Israel National Air Monitoring System. The data were recorded every 5 min by a dichotomous ambient particulate monitor (Thermo Scientific 1405DF; Thermo Fisher Scientific Inc.) that utilizes two tapered element oscillating microbalances (TEOMs) to provide a continuous, direct mass measurement of particle mass. Other data recorded by the station include the levels of major air pollutants (CO, SO_2_, NO_2_, and O_3_) and meteorological variables (air temperature, relative humidity, wind speed and directions, solar radiation).

During strong dust events, daily outdoor PM_10_ concentrations can exceeded 2000 µg m^−3^ while hourly concentrations were as high as 5000 µg m^−3^. In this study, real-time measurements were related to strong dust storms (Figure 1). PM_10_ data were used in order to identify and select strong dust events based on the net contribution of PM_10_ concentrations to the area’s background levels [8]. Four storm levels are defined: low = 264 µg m^−3^; medium = 661 µg m^−3^; high = 1322 µg m^−3^; severely high = 1983 µg m^−3^. The strong dust storm of 11 February 2015 was at the focus of this study.

Particle characteristics. The chemical and physical properties of the atmospheric dust sample collected during the event of 11 February 2015 was analyzed. The sample was collected on a building roof within the campus of Ben-Gurion University of the Negev using settled dust collectors [2]. The sterilized settling-dust collectors consist of a tray filled with layers of glass/quartz marbles. Atmospheric dust particles that cross the tray aperture are trapped in the marble matrix due to the matrix cohesion and roughness along with reduced air speed near the surface of the tray. At the end of the event the dust sample (~5 g) was moved into sterilized glass vials for size distribution and elemental analyses. Particle size distributions over the range of 0.08 to 2000 μm were obtained by high-resolution laser diffractometer (ANALYSETTE 22 MicroTec Plus) [19,20]. Each sample was dispersed by sonication (at 38 kHz) in a Na-hexametaphosphate solution (0.5%), then transferred to a fluid module of the instrument (containing deionized water), and subjected to 3 consecutive 1 min runs at a medium pump speed of 6 L min^−1^. The data were processed using the Mie scattering model (RI = 1.56, AC = 0.01) with an error < 5.0%. The MasControl software (Fritsch, Idar-Oberstein, Germany) was employed to determine the following relevant parameters: mean size, median, modes in multiple modal distributions, sorting values, and size fraction weights. Elemental analyses were done using a wavelength dispersive X-ray fluorescence spectrometer. Quantitative analysis of the detected elements was done using Omnian software (Axios, Panalytical Co., Malvern, United Kingdom).

Real-time PM measurements. Continuous real-time measurements were recorded simultaneously in indoor and outdoor urban environments in five households located in the center of Beer-Sheva (Figure 1). The selected households were apartments characterized by common building materials and construction (concrete-based) of Beer Sheva. The apartments were at the middle floors in their building (3–5 stories) to avoid possible disturbance because of proximity to a yard (first floor) or a roof (last floor). The indoor measurements at these houses were continued for a duration of about three hours during the dust event. The measurements were obtained with portable dust monitoring devices DustTrak DRX 8534 (TSI, Shoreview, Minnesota, USA); reading resolution of 0.001 mg m^−3^, accuracy of 1%) that recorded both PM_2.5_ and PM_10_ data and that were situated at each indoor and outdoor measurement point.

In the indoor environments, the dust monitoring devices were operated in the main living area of each house at 1 m height above the floor (with no carpet). The PM measurements were performed under conditions of minimal human interference during the dust event (closed windows, air conditioning not operating, no cooking). The corresponding outdoor PM_2.5_ and PM_10_ were recorded by another DustTrak DRX 8534 situated on the balcony with overhead, which is at the height of the apartment floor. The monitor was placed in north-east side balconies to avoid direct wind during the dust event (south-west) and keep measurement stability. DustTrak monitors were factory-calibrated for a respirable particle fraction based on the Arizona Test Dust standard (i.e., ISO 12103-1, A1 test dust), which is representative of a wide variety of aerosols; they generate a relatively accurate measure of PM_2.5_ and PM_10_ concentrations during a dust storm compared with the TEOMs as demonstrated in Jayaratne et al. [21] and Krasnov et al. [9].

The dust event was examined by satellite images (MODIS Level 1 and VIIRS Level 1). The air mass transport in the region was obtained through Backward Trajectories model (NOAA/ARL HYSPLIT-4) prior and during the event for three different altitudes (500, 1000, and 1500 m above ground level). This revealed synoptic condition of a cold low-pressure system with the Cyprus Low that is most prevalent in the winter season [8].

Means and standard deviations were calculated with the aim to describe the outdoor and indoor PM concentrations during the dust storms. Spearman correlation coefficient was used for univariate analysis of indoor and outdoor concentrations. For statistical analysis SPSS (SPSS Inc., Chicago, IL, USA) was used. Statistical significance was taken as *p* ≤ 0.05.

## 3. Results

Figure 1 shows the daily PM_10_ concentrations as recorded by the environmental station in Beer-Sheva for the last 15 years. Overall, there were 679 natural dust days, i.e., when the average daily PM concentration exceeded the calculated threshold value (71 µg m^−3^), below which days were classified as clear, non-dust days (see details in reference 14). In addition, daily PM_10_ concentrations during eleven storms exceeded 661 µg m^−3^ (dashed line in Figure 1), which we defined as strong events (which include events with high to severely high PM_10_ levels).

**Figure 2:**
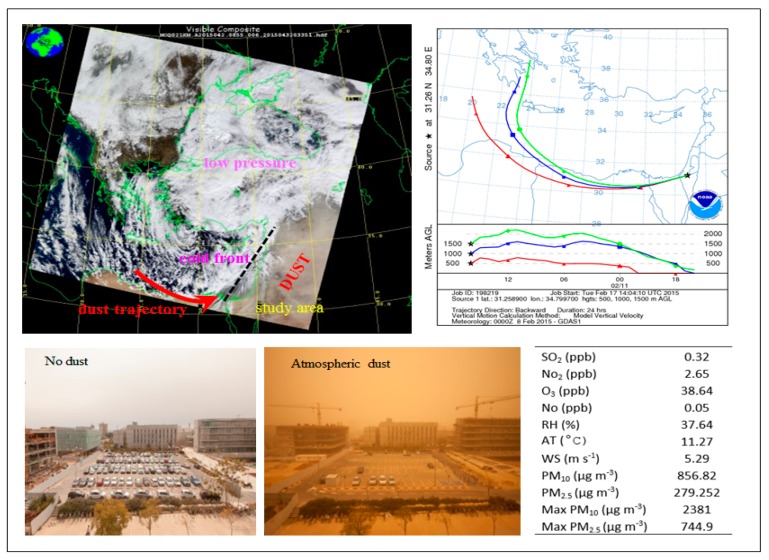
Satellite images (MODIS) of the study region during the dust storm of 11 February 2015, along with air mass transport at different heights above ground level (AGL). In the lower panel: daily recorded averages of major meteorological variables and pollutants measured in the environmental monitoring station of Beer Sheva during the dust event of 11 February 2015. AT—air temperature; RH—relative humidity; WS—wind speed; PM—particulate matter. The two photos were taken in the same location, the campus of Ben Gurion University (Beer Sheva), during a non-dust day (left) and during the dust event of February 2015 (right) in the study area.

A recent dust event that occurred on 11 February 2015, which is a typical strong event of the 15-year period studied (but not the strongest one), was explored in detail to represent the phenomenon in question. The environmental conditions during the event of 11 February 2015 are presented in Figure 2. Satellite images of the study area are shown together with air mass trajectories (Hybrid Single-Particle Lagrangian Integrated Trajectory model [22]) for three different altitudes (500, 1000, and 1500 m above ground level).

**Figure 3:**
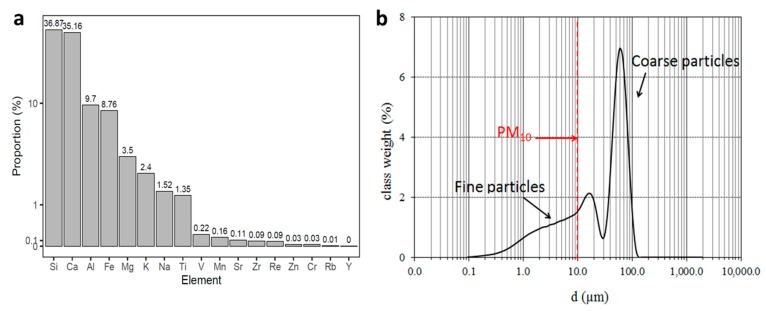
Chemical and physical characteristics of dust samples collected during the event of 11 February 2015. Elemental composition by X-ray fluorescence spectrometer on logarithmic Y-axis (**a**), and particle size distribution by laser diffractometer (**b**).

The physical and chemical properties of the dust samples during the 11 February 2015 event were analyzed. Elemental analysis showed that dust sample compositions were typical of dust from this region [2,23]. The most common minerals were Si from soil minerals and Ca, which can be found mainly in sedimentary environments and in the calcareous soils and rocks characteristic of the arid land of the Mediterranean basin (Figure 3a). The dust samples are characterized by a bi-modal distribution of particle sizes. The cutoff is 18 µm (Figure 3b), which is the size used to distinguish between fine (from distal source) and coarse (from local source) fractions.

**Figure 4:**
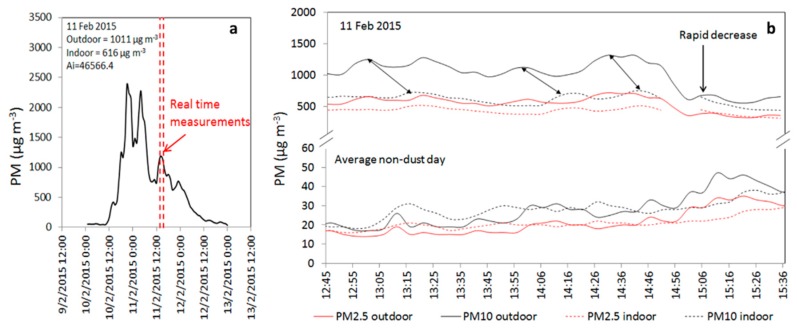
(**a**) The recorded PM10 concentrations by the environmental monitoring station in Beer-Sheva during the dust storm in 11 February 2015. The average PM_10_ concentrations and storm intensity (AI) are presented.) (**b**) Real-time measurements of hourly indoor and outdoor variations in PM_10_ and PM_2.5_ recorded (*n* = 30) in the houses in Beer Sheva compared with the concentrations recorded in the houses during non-dust days. The arrows between the PM_10_ curves represent the time lag between the indoor and outdoor concentrations.

The real-time measurements by portable dust monitors were used to study the response of indoor PM_10_ and PM_2.5_ to those of the outdoor during the dust event of February 2015. The results, shown with a comparison to a typical non-dust day in the study area (Figure 4), represent the afternoon hours, when PM concentrations were typically at their highest levels during the daily dust cycle. The outdoor PM_10_ concentrations measured by the portable device are consistent with the data recorded by the monitoring station situated at a central location in Beer-Sheva. Outdoor PM_10_ concentrations were above 1000 µg m^−3^ for PM_10_ and above 500 µg m^−3^ for PM_2.5_ (Table 1) compared to a non-dust day, when PM_10_ levels reached a maximum of 47 µg m^−3^ and PM_2.5_ levels were 31 µg m^−3^. The simultaneously measured indoor levels fluctuated in parallel with the outdoor levels but with a time lag of about 15 min for the peak of PM_10_ (see arrows in Figure 4). The response of PM_2.5_ was less consistent with no specific time lag.

The ratio of indoor-to-outdoor concentrations (I/O) may provide insight into the relative contributions of the different PM sources to the indoor concentration levels. The calculated I/O for the houses during the non-dust days is equal to 0.94 ± 0.20 for PM_10_ and 0.93 ± 0.13 for PM_2.5_. The I/O during 11 February 2015 was 0.61 for PM_10_ and 0.74 for PM_2.5_ (Table 1). The decrease, and a much smaller than 1.00 ratio of I/O, indicate the absence of indoor PM sources and, therefore, the overwhelming contribution that the particles from outdoors make on the indoor concentration levels.

**Figure 5:**
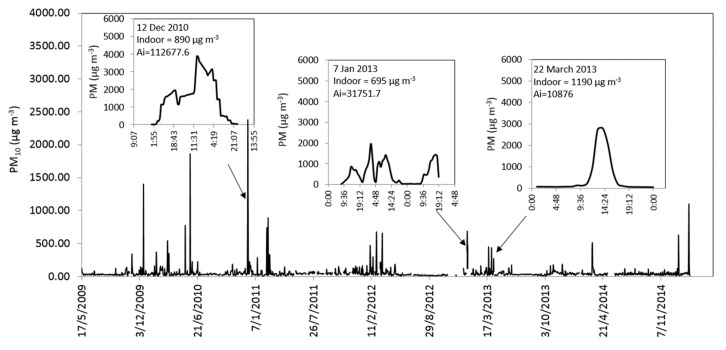
Outdoor PM_10_ levels and average indoor PM_10_ concentrations during strong dust storms recorded in the northern Negev from 2009 to 2014. The parameter Ai represents storm intensity [8].

**Figure 6:**
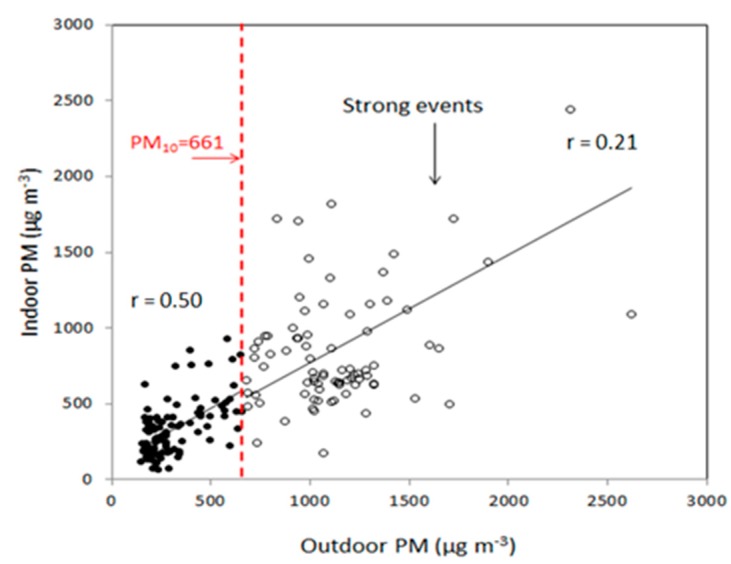
Correlation between outdoor and indoor PM_10_ concentrations during the different PM levels. Strong dust storms are distinguished by their PM levels that exceed the calculated threshold of 661 µg m^−3^.

Because dust storms are common phenomenon not only in the southeastern Mediterranean, but also in many other regions around the world, it is essential that we improve our understanding of their potentially detrimental effect on the indoor environment. We present here three strong dust events (12 December 2010, 7 January 2013, 22 March 2013) as additional cases on the impact of dust on indoor concentration (Figure 5). The events are presented with hourly indoor PM_10_ concentrations together with their average concentrations and event intensity (Ai). The Ai was calculated on an hourly basis as the area under the storm curve [8,14]. The results clearly show the differences in dust storms in terms of hourly PM levels and event durations. Figure 6 presents the correlation between outdoor and indoor PM_10_ concentrations for a wide range of dust concentrations from different events. The total correlation between indoor and outdoor measurements for all storms is 78%. For storms with concentrations below the calculated threshold of strong events (661 µg m^−3^), the correlation is 50% while for higher concentrations the correlation is only 21%.

## 4. Discussion

The conditions of the dust event that occurred on 11 February 2015 are shown in Figure 2. The average wind direction (261°) was consistent with the NOAA HYSPLIT Trajectory Model (Boulder, Colorado, USA). The relatively strong winds measured at 10 m height (average speed of 5.3 m s^−1^) and the low air temperatures were associated with the presence of a low-pressure synoptic system in the eastern Mediterranean and of a cold front to the west. The daily averages of major meteorological variables and daily average levels of major pollutants during the dust event are displayed in Figure 2. With the exception of PM_10_ and PM_2.5_, air pollutant levels were not affected by the dust event and remained relatively low. The daily PM_10_ concentrations, however, were about 20-fold higher than the background value of the area on non-dusty days (71 µg m^−3^). The possible sources of the finer particles (Figure 3), which can be carried longer distances, are from soils along the North-African–Sinai–Negev path (see mass trajectories in Figure 2). Particles that are smaller than 10 µm constituted only 1.53% of the total suspended dust volume, but the overall result is high atmospheric PM_10_ concentrations and air pollution. Stormy days in this region engender dramatic atmospheric changes, clearly manifested in the “cloudy” yellowish appearance and corresponding reduced visibility associated with dusty days (Figure 2)

The results of the real-time measurements of indoor and outdoor PM_10_ and PM_2.5_ during the dust event of February 2015 are presented in Figure 4. The indoor concentrations, which peaked at 700 µg m^−3^ for PM_10_ and 470 µg m^−3^ for PM_2.5_ at their peaks, remained high as long as the outdoor concentrations were high. During non-dust days, indoor concentrations were, on average, 30 µg m^−3^ for PM_10_ and 20 µg m^−3^ for PM_2.5_, indicating that these values increased by more than 20 fold during dust events. However, the indoor PM_10_ levels did not decrease at the same rate as the outdoor levels, meaning that due to dust events, indoor air quality stays dusty for a longer period than the outdoor air due to dust events. Although quantitative information on how long until PM_2.5_ and PM_10_ levels returned to baseline levels (>30 µg m^−3^ for both PM) is not presented in this study, it can be assumed that the baseline will be reached only after several hours considering the settling velocity of dust particles that are < 10 µm [1]. This finding is also supported by the strong correlation for the event of February 11 between the indoor and outdoor concentrations for both PM_10_ and PM_2.5_ (*r* = 0.70). The higher I/O ratios of PM_2.5_ compared to those of PM_10_ are related to the more efficient infiltration of the houses by the fine outdoor particles due to their relatively smaller sizes [10].

The weaker correlation coefficient observed for the strong events (Figure 6) can be explained by dust storm behavior over time. As shown in Figure 4, the indoor levels did not decrease at the same rate as the outdoor concentrations mainly because of the dependence of the airborne particles that accumulated indoors on the specific air exchange rates of the building that they infiltrated.

The main study limitations refer to the number and the representation of the measured households. In this study, continuous real-time measurements were recorded simultaneously in indoor and outdoor urban environments in five households located in the center of Beer-Sheva. Using more households will obviously strengthen the database and the significance of the results. However, such a study design requires a large set of PM monitors, which are expensive. Nonetheless, the recent trend of developing and using commercial off-the-shelf (COTS) sensors [24] may be a more plausible economic solution for the next generation of air quality studies.

## 5. Conclusions

The main contribution of this study is to show how the indoor air quality is very sensitive to changes in atmospheric PM_10_ concentrations in an urban environment that is subject to strong dust events. The conclusions revealed from the results of this study are: (1) the indoor PM_10_ and PM_2.5_ concentrations increased by more than 20-fold during the dust event. (2) The indoor levels peaked at about 700 µg m^−3^ (PM_10_) and 470 µg m^−3^ (PM_2.5_) and fluctuated in parallel with the outdoor level, but with a time lag of about 15 min. (3) The I/O ratio emphasizes the outdoor effect, while indoor PM levels tend to remain high for longer periods than the corresponding outdoor levels. (4) The correlation between indoor and outdoor is higher in moderate dust events, which are more frequent.

This extreme spike typical in indoor PM_2.5_ and PM_10_ levels during dust events characterized by high outdoor PM levels is assumed to be even higher in locations with poor housing conditions. These findings suggest that considerations about potential dust exposure should be an integral part of the planning and building stages. Further studies are needed to provide more quantitative information about air pollutants in urban environments, and their spatial and temporal distributions, using advanced technology of small sensors in the perspective of smart cities.

## Figures and Tables

**Table 1:** Statistical description of particulate matter (PM) concentrations (µg m^−3^), and the ratio of indoor-to-outdoor (I/O) during the dust event based on the real-time PM record with time interval of 5 min (*n* = 30). (SD—standard deviation).

	Mean	Median	Maximum	Minimum	SD	I/O Ratio
PM_10_ outdoor	1011.2	821.1	2277.2	323.4	320.1	N/A
Indoor 1	773.2	682.3	835.3	365.2	223.4	0.76
Indoor 2	620.1	560.6	790.2	371.8	185.6	0.61
Indoor 3	590.3	430.3	683.3	254.1	175.5	0.58
Indoor 4	433.8	395.9	495.6	244.0	120.5	0.43
Indoor 5	665.3	480.5	695.4	349.3	188.6	0.66
Average indoor (1–5)	616.6	509.9	699.9	316.9	178.8	0.61
PM_2.5_ outdoor	529.5	393.3	744.9	106.8	252.2	N/A
Indoor 1	501.2	421.3	488.2	212.3	233.5	0.95
Indoor 2	410.8	415.9	548.0	235.6	165.8	0.78
Indoor 3	403.2	371.2	471.2	162.6	215.2	0.76
Indoor 4	272.5	261.4	352.6	120.3	80.3	0.51
Indoor 5	375.4	351.0	491.1	198.6	133.0	0.71
Average indoor (1–5)	392.7	364.2	470.2	185.9	163.6	0.74
